# Exploring mHealth applications for self-management of chronic low back pain: A survey of features and benefits

**DOI:** 10.1016/j.heliyon.2023.e16586

**Published:** 2023-05-26

**Authors:** Saba Kheirinejad, Aku Visuri, Sharadhi Alape Suryanarayana, Simo Hosio

**Affiliations:** University of Oulu, Oulu, Finland

**Keywords:** Mobile health, mHealth, Chronic low back pain, CLBP, Self-management, mHealth solutions, mHealth applications, Smartphone applications, Mobile applications, Mobile apps, Health professionals, Patients

## Abstract

The adoption of Mobile Health (mHealth) for self-management is growing. mHealth solutions are commonly used in public healthcare and health services, where they are appreciated for their ease of use, broad reach, and wide acceptance. Chronic Low Back Pain (CLBP) is one of the most common health problems and a leading cause of disability. As such, it imposes a tremendous burden on patients and society. Studies have proposed that mHealth self-management solutions, such as mobile applications, can supplement traditional care methods and benefit patients, particularly in self-managing CLBP easier. To this end, the number of available mobile applications for CLBP has increased. This paper i) provides an overview of scientific studies on mobile applications for CLBP management from three different viewpoints: researchers, health professionals, and patients, ii) uncovers the application features that were seen as beneficial in the studies, and iii) contrasts the currently available applications for CLBP in Google Play Store and Apple App Store against the discovered features. The findings show that “Personalization and customization” is the most significant feature as it is beneficial from stakeholders' viewpoint and is represented by most applications. In contrast, “Gamification” and “Artificial intelligence” are the least significant features, indicating a lack of attention from application creators and researchers in this area.

## Graphical abstract

**Figure:**
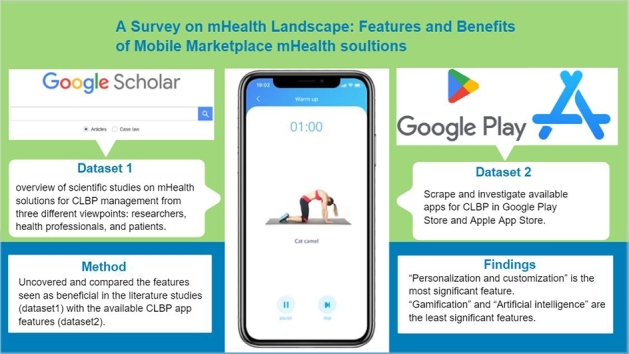

## 1 Introduction

The global adoption of Mobile Health (mHealth) is growing due to decreasing hardware costs and the increasing amount of mobile devices such as smartphones, tablets, and wearable devices in circulation [1]. mHealth solutions are commonly used in public healthcare and health services, where they are appreciated for their ease of use, broad reach, and wide acceptance [2]. Smartphones have become an integral part of people's everyday lives [3], and mHealth applications are also almost available 24/7 and do not have geographical limitations for people from remote and rural areas [1], [4], [5]. They could even benefit vulnerable people more in self-managing their pain without being bothered and wasting time and money [6]. Self-management is defined as “*the ability to manage the symptoms, treatment, physical and psychological consequences, and lifestyle changes in people with chronic conditions*” [7], [8], [9]. In recent years, self-management has been suggested as a potential supplementary aid for people with Low Back Pain (LBP). LBP is one of the most common health problems and one of the leading causes of disability, which burdens patients tremendously. LBP lasting over three months is defined as Chronic Low Back Pain (CLBP) [10]. The typical self-management procedures for CLBP mainly consist of suitable exercises, followed by health education and professional advice [11]. Also, mHealth application-based self-management program accompanying conventional physiotherapy has shown promising results in field studies [11]. To better understand how to support self-management decision-making, more research is needed to explore the factors that patients and health professionals consider when using and prescribing mHealth applications [12].

Studies indicate that involving medical experts in designing and developing mHealth applications is a determinant factor for their endorsement and support [13]. It also makes those applications more valid [14]. However, many applications in different marketplaces have been designed without the involvement of health professionals [15]. However, the development of mHealth applications for CLBP should encompass a multidisciplinary approach between application creators, researchers, healthcare professionals, and patients [16]. To create practical applications, creators need to learn from stakeholders. Research can help them identify features to benefit various stakeholders more efficiently [17].

In this study, we first review the relevant scientific literature to identify mHealth application features and their benefits for self-managing CLBP from the viewpoints of three stakeholders: 1) patients, 2) researchers, and 3) health professionals. Second, we scrape and analyse 163 available mobile applications from the Google Play Store and Apple App Store using keywords related to CLBP to investigate which features identified in the first step are most commonly offered to users. We introduce a novel metric named “*benefit score*” to represent a given application feature's aggregated perceived benefits from all stakeholders' viewpoints (patients, researchers, and health professionals). We use the benefit score to cross-evaluate which features are either overrepresented or underrepresented in modern CLBP applications from the lens of scientific mHealth literature. Eventually, we cluster the applications to understand what features usually come along with each other in the applications and estimate the significance of each cluster.

Overall, this study will mainly help mHealth application creators to know the benefits of different features from various stakeholders' perspectives and create more beneficial applications without putting extra effort into less beneficial features. Furthermore, researchers, health professionals, and patients will also benefit more from applications that have been scientifically created.

## 2 Related work

### 2.1 Chronic low back pain (CLBP)

CLBP is the leading cause of disability worldwide, spanning across all age groups and geographies irrespective of income levels which imposes a tremendous burden on patients [18]. CLBP is a common reason for doctor visits and absence from work [19]. CLBP is one of the most commonly reported chronic pain conditions [20], and it has been the main contributor to overall years lived with disability [21]. The prevalence of CLBP steadily increases from the third decade of life until the age of 60 [22], with a higher incidence in women [22], [23]. Many people with CLBP have various problems in which psychological, social, and biophysical factors, comorbidities and pain-processing mechanisms affect both the pain experience and the associated disability [18].

Nowadays, a sedentary lifestyle has become widespread as an increasing number of individuals spend extended periods in a seated position at work and during leisure time [24]. Exercise is an efficient way to ease stiffness and pain, build up muscle, and improve strength, stamina, flexibility, and general fitness [25], [26]. Exercise keeps the back moving by stretching tight muscles and joints and stops the spine from seizing up [27], [28]. Staying active will help one get better faster and prevent more back trouble [29]. Exercise is relatively helpful in managing CLBP, and adherence to exercise might be enhanced if participants are engaged. Identifying factors that improve engagement would enable clinicians to prescribe suitable solutions [30].

### 2.2 mHealth solutions for managing CLBP

The World Health Organization (WHO) Global Observatory for eHealth (GOe) defines mHealth *“as medical and public health practice supported by mobile devices*” [31]. mHealth solutions refer to the broad range of mobile health technologies designed to improve health outcomes and promote healthy behaviours. These solutions can include hardware and software components and may incorporate sensors, mobile devices, and communication technologies. mHealth solutions can be used to diagnose and treat a wide range of health conditions, as well as to improve overall health and wellness through behaviour change and health education [32]. mHealth solutions have become more accessible worldwide [33]. Purposes such as helping people to succeed in weight management, stress management [34], encouraging and monitoring behaviour change, self-diagnosis, or rehabilitation schedule management [35], and self-monitoring of chronic health conditions, medicine adherence reminders have emerged. mHealth can also help provide suitable exercises to improve chronic pains [36], and direct interactions with the healthcare system [37] are common. mHealth may also enable meaningful information exchange between consumers and healthcare professionals by leveraging, collecting, and distributing electronic records, patient data, remote monitoring, and prescriptions. Many fitness and wellness applications can also provide supplementary data to caretakers [38].

There is evidence in medical literature for the efficiency of mHealth solutions in treating CLBP. By conducting a PubMed search, Cavanagh et al. [39] showed that telehealth, internet-based programs, and mobile applications have been the most promising technologies in recent years. The mentioned solutions reduce pain symptoms and facilitate self-management, anonymous participation, and portable and cost-effective treatment. Patients with CLBP who experience recurrent pain and disability after treatment are prone to seek extra care, including medication, physiotherapy, emergency department attendance, specialist consultation, or spinal surgery [40].

Amorim et al. [41] conducted a pilot randomized controlled trial with blinded outcome assessment. They used physical activity information booklet, one face-to-face and 12 telephone-based health coaching sessions supported by an internet-based application, and an activity tracker (Fitbit) as the interventions. They suggested that the health coaching physical activity method is possible and well accepted by participants and may decrease care-seeking in CLBP patients after treatment discharge. Chen et al. [42] showed that using mHealth and routine care solutions simultaneously has better efficiency than alone routine care in disability and decreases pain intensity in patients with CLBP. In addition, they revealed that using telephone calls as a mHealth intervention might positively reduce pain intensity and disability.

Schaller et al. [43] provided movement coaching, including face-to-face intervention, telephone, and internet aftercare to an intervention group. Bailey et al. [44] evaluated the efficiency of a 12-week digital care program in a large population of patients with chronic knee and back pain. Participants showed high completion and engagement rates. A significant positive relationship between engagement and pain reduction was recognized, a finding not previously demonstrated in a digital care program. Pfeifer et al. [4] did a systematic review and meta-analysis to investigate the efficacy of mobile application-based solutions for chronic pain on pain intensity. They showed emerging evidence that mobile applications could reduce pain among non-cancer pain patients.

Previous research has demonstrated that involving medical experts in designing and developing mHealth technologies is a determinant factor for their endorsement and support. It leads to making those technologies more valid [14]. However, integrating mHealth solutions in clinical practice is a complex challenge. Vecchia et al. [45] investigated the willingness of French healthcare professionals to prescribe mHealth solutions for their patients. They showed that healthcare professionals are inclined to fully integrate mHealth solutions into their practice, especially if they can access tools to guide them and show their way in digital health. For instance, the tools provide information about the benefits, pros, cons, and how mHealth solutions are developed, validated, and certified. Mutebi et al. [46] investigated the perceptions of 23 family physicians in Belgium on using mHealth for primary care, such as monitoring and data collection in chronic diseases, via an online questionnaire. They found that less than half of the physicians were familiar with the mHealth term, and there is resistance to mHealth use among them. Indeed, most physicians showed a lack of interest in delivering such services.

### 2.3 mHealth applications for managing CLBP

Studies indicated that mHealth self-management methods, like mHealth applications, could help with traditional approaches to better managing CLBP [11]. Especially since the beginning of 2020, COVID-19 has become prevalent globally, and its impact might last until 2025 [47]. This period has led to the rapid development of mHealth solutions [48]. mHealth applications are software programs designed to run on mobile devices such as smartphones and tablets. These applications are intended to provide health-related services, such as tracking health data, providing health education, or allowing patients to communicate with healthcare providers remotely [49]. The applications could include different types of features to help the patient's improvement, such as exercise program (biomechanical, aerobic, mind-body, or a combination of three previous categories), manual therapy (manipulation, mobilisation, or soft tissue techniques such as massage), materials for educating the patients, a variety of physical and psychological treatment, etc. [50]. There are many applications for self-management of CLBP in the Google Play Store and Apple App Store. mHealth applications are easy to implement, cost-effective, and help patients become more independent in managing their pain. In addition, application-based solutions are almost available 24/7 and do not have geographical limitations for people from rural or remote areas [4] unless specified by application vendors. However, their effectiveness in improving patients' conditions has not been rigorously evaluated, and users have no clear guidance on choosing practical and high-quality applications. To ensure that application contents are correct, evidence-based, and fascinating, application creators must work closely with researchers, healthcare professionals, and patients [51].

Approaches including education, advice, and a significant focus on self-management, such as lifestyle behavioural change, physical activities, and medications as required, could be adopted to lift the burden of the cure of musculoskeletal pains like CLBP off the clinicians and help the patients self-managing their pain [52]. Different interventions help CLBP patients adhere to physiotherapists' prescriptions to self-manage their pain, such as activity monitoring and feedback system, written exercise instructions, behavioural change program with booster sessions, and goal setting [53]. Physical activities play a fundamental role in the prevention and rehabilitation of CLBP. However, adherence to physical activities in daily life is a common problem for patients. Thus, many mHealth applications add features such as reminders, rewards, and gamification to increase adherence to physical activities. Monitoring is crucial in chronic diseases but unclear in musculoskeletal pain conditions like CLBP. Pain awareness might be helpful; however, concentrating on the pain might have adverse effects. Yet, Kongsted et al. [54] did not find any evidence that frequent self-reporting of pain negatively impacts CLBP patients. On the contrary, they showed that it is helpful. Studies suggest that a good partnership between patients and health professionals positively affects the self-management ability of CLBP patients [55].

mHealth applications can be prescribed as practical self-management tools for patients. However, it would be challenging for physicians to search many mHealth applications to find the right ones to recommend. Byambasuren et al. [56] evaluated the current knowledge and use of mHealth applications for the 1014 general practitioners in Australia. The general practitioners showed that a lack of understanding of practical applications and trustworthy sources of informative applications are the most critical barrier to their application prescription. Therefore, the authors suggested that a curated compilation of practical mHealth applications or an application library specifically aimed at health professionals would help solve both barriers. Dahlhausen et al. [57] investigated physicians' and psychotherapists' attitudes toward mHealth applications, barriers to adoption, and potential treatments across Germany. Health professionals showed that mHealth applications had improved patient care and treatment success. Moreover, they expressed that insufficient knowledge and information and reimbursement of medical services are the most critical barriers to prescribing the applications. Jezrawi et al. [12] studied 133 physicians' viewpoints on selecting, recommending, and evaluating mHealth applications in Canada. They showed that personal opinion is the essential evidence physicians use to support their recommendations. Moreover, they showed that an unbiased, standardized consensus panel review by topic experts is the crucial level of evidence that they would prefer to have available on health applications before recommending or prescribing them to patients.

Other systematic and standardized methods beyond the “star rating” that help the researchers, health professionals, and end-users about the quality of an application or its medical evidence are mainly missing. To this end, Stach et al. [58] created a database called *Mobile Health App Database (MHAD)* that uses an instrument named *The Mobile App Rating Scale (MARS)* which categorizes the purpose and function of mHealth applications and evaluates their quality. Machado et al. [51] searched the Australian Apple App Store and Google Play Store for CLBP self-management applications to investigate their content and quality to help consumers make better choices. They used the *2016 National Institute for Health and Care Excellence (NICE)* guidelines to specify whether interventions recommended by the included applications were evidence-based. Then they used the MARS [59] to rate the quality of applications that recommended evidence-based solutions. Didyk et al. [60] investigated 25 applications about CLBP and sciatica from Google Play Store and Apple App Store. They measured the quality of applications using MARS and NICE. They indicated that mobile applications could improve outcomes for people with CLBP aligned with current self-management guidelines.

Hosio et al. [61] proposed an application named *Back Pain Buddy* to educate people with CLBP and collect contextual data. The result indicated that people are keen to donate their data. Coe-O'Brien et al. [16] selected and downloaded 74 free applications for CLBP from Google Play and App Store to evaluate their quality using MARS. They also examined the outcome measures used and assessed the outcome measures against the International Classification of Functioning, Disability, and Health (ICF) core set classifications for CLBP. They showed that the overall quality of the applications is relatively low, and only 5% of the applications utilize valid outcome measures. The review also highlighted that the few outcome measures used in the applications do not represent all the four core CLBP criteria set by ICF. Escriche-Escuder et al. [62] selected 17 applications related to CLBP from the Google Play and App Store in Spain and the United Kingdom. Again, they measured the quality of the applications using MARS. They showed that the assessed applications generally had good quality, especially in functionality and aesthetics. However, they must enhance factors such as engagement and information to increase their effect on users and ensure better security and privacy. Effectual cooperation between researchers and industry is required to create better user-centred mHealth applications to empower patients with these conditions. Carvalho et al. [63] selected ten mHealth applications related to spine disorders from the Google Play and App Store in Brazil. Two independent reviewers evaluated the applications' quality using MARS. They showed that the quality of the applications could be better, and most scored poorly for credibility, user interface, and engagement.

## 3 Study design and datasets

Our study methodology consists of the collection and cross-comparison of two datasets. We first conduct a literature review on how mHealth applications can help manage CLBP, collect a set of features related to mHealth applications, and connect each feature to a list of one or more benefits that those features provide according to the literature. We then investigate the current mobile application marketplaces for applications related to CLBP management. We manually categorize those applications according to the features they provided and according to the feature set from our initial investigation of related work.

Through the described comparison, we seek to highlight gaps in current mobile marketplaces and show discrepancies between what mHealth features experts see as beneficial and where mHealth application creators typically put the most effort. In the following sections, we will first explain the methods and results from the literature review and then the process of searching and obtaining information about mobile application marketplaces.

### 3.1 Perspectives on features provided by mHealth applications and their benefits

We searched Google Scholar to find studies investigating different perceptions from various stakeholders using mHealth solutions for self-managing their chronic pain, such as CLBP. In the following sections, we have divided the findings into the perspectives of three distinct entities; the researchers, health professionals, and patients. The definition of the terms are as follows,

• Researchers' viewpoint: The papers in which researchers investigated various mHealth solutions created for self-managing CLBP.
• Health professionals' viewpoint: The papers in which health professionals were asked about their perception of using mHealth solutions to support people with CLBP to self-manage them.
• Patients' viewpoint: The papers in which patients were asked about their perception of using mHealth solutions for self-managing their CLBP.

#### 3.1.1 Methodology

We conducted our literature using a free-form method described in the following using the PRISMA [64] annotation. We did not keep a comprehensive records tally at each stage of the process, only the result.

We used Google Scholar as our records database and conducted manual searches using a combination of keywords in detail in Table 1. Each search included one keyword from each group, resulting in search strings like “medical experts viewpoint mHealth pain management chronic low back pain”. We iterated this process systematically (picking keywords from each category) multiple times until saturation, i.e. no new papers surfaced in the search results. A researcher initially screened each result (title, abstract) and chose to be included or excluded. If an article was not available through open access or our university, it was also excluded. The included articles were then retrieved for further analysis.

**Table 1:** Keywords and keyword groups used in our literature search process.

Group	Keywords
Perception	“viewpoint”, “point of view”, “standpoint”, “perception”, “perspective”, “attitude”
Stakeholders	“health professionals”, “medical experts”, “clinicians”, “physicians”, “doctors”, “practitioners”, “physiotherapist”, “researchers”, “patients”, “users”
Technology	“applications”, “apps”, “mHealth”, “mobile health”, “mobile app”, “smartphone app”, “mobile technology”, “health technology”
Method	“self-management”, “pain management”, “intervention”, “solution”
Condition	“chronic low back pain”, “low back pain”, “back pain”, “CLBP”, “chronic condition”, “backache”, “lumbago”, “lumbar”, “spinal pain”, “pain”, “chronic pain”, “posture”

Each retrieved article was then assessed for eligibility for our purposes, and articles were excluded for various reasons; some articles focused solely on the technical development of mHealth applications did not discuss either features or benefits or were otherwise out of scope for our purposes. Articles that did not examine the attitudes of researchers, healthcare professionals and patients towards utilizing mHealth solutions for the purpose of self-managing chronic pain were not included. The ultimate decision was made by manually reading the article's results and conclusions. Finally, each relevant citation in each article was added to our list of records for retrieval.

This process yielded 35 research articles directly related to our study purposes and highlighted both features of mHealth applications and their benefits. In Table 2, we provide a comprehensive explanation and definition of the various features offered by mHealth applications.

**Table 2:** Explanation and definition of features provided by the mobile applications.

Feature	Description
Activity tracking	Automated monitoring and tracking of physical activity, walking, running, and calorie consumption.
Physical activity	Instructions for physiotherapy activities, stretching, extension exercises, and workouts.
Personalization and customization	Provides personalized instructions and plans with customization options.
Yoga/meditation	Contains yoga, meditation, mindfulness and breathing exercises.
Education/advice	Information and knowledge in video, audio, or text format about chronic pain and how to manage it.
Scheduling and reminders for exercise or behavioural change	Providing reminders for doing physical activity, taking medication, correcting the back postures, etc.
Goal setting	Provides methods to set goals to reach particular physical activities, a particular diet, lose weight, etc.
Diary/self-assessment	Providing features for recording the pain, sleep, diet, etc., in daily surveys or diaries.
Gamification	Application contains gamified features or rewards to encourage people to continue using the application.
Patient-physician partnership	Features that facilitate the communication between patients (users) and physicians. Features such as chat, call, video call, sending the data and statistics to the physicians, etc.
Social support	Social features to facilitate communication between patients (users), their peers, and their support networks, such as friends and family members, to share knowledge, encourage each other to better self-manage, and increase empathy.
Artificial Intelligence (AI)	AI refers to integrating machine learning algorithms, computer vision techniques and natural language processing into mobile applications to enable them to perform complex tasks, learn from user interactions, offer personalized experiences, provide user preferences recommendations, and automate tasks such as scheduling and reminders.

#### 3.1.2 Researchers' viewpoint

This section highlights the examination of various mHealth applications developed for the self-management of CLBP in relevant studies from the researchers' viewpoint. This section will delve into the details of the selected papers, outlining the specific mHealth applications studied and the outcomes reported. By doing so, we aim to contribute to the current understanding of the efficacy of mHealth applications in managing CLBP.

Chhabra et al. [65] investigated the effect of using a smartphone application called *Snapcare*. They showed that combining an engaging interface with individualized goal-setting is likely the most efficient method for increasing physical activity levels and adherence to home exercise programs. Accordingly, patients who used the Snapcare application found reaching their daily physical activity goals stimulating and rewarding. Zheng et al. [66] provided exercise prescription video, guidance, and educational content to the participants with the *Ding Talk* application. They showed that mHealth-based exercise (via guidance) is a valuable and efficient way to manage CLBP. Moreover, they indicated that guidance plus education is more effective in reducing short-term negative emotions and improving treatment adherence than guidance only. A retrospective study demonstrated that in a pre-selected population, a digitalizing multidisciplinary rehabilitation application *Kai* including patient education, video-guided physiotherapy, and mindfulness training for the self-management of CLBP, reduced user-reported pain levels significantly [67]. Dobija et al. [68] conducted a review to identify recent areas of mHealth application usage for managing CLBP. They divided the primary use of mobile applications into four categories: self-management, telerehabilitation, evaluation, and data collection. They found that self-management is the most-studied use. Although, they cautioned about monitoring the consequence of smartphone-related compulsive behaviour. Agnew et al. [69] did a review to investigate the use of mHealth in musculoskeletal physiotherapy. They showed that mHealth is useful in adherence to treatment and can potentially be as productive as regular physiotherapy while being more cost-effective. They found that patients most widely accepted communication with a clinician via telephone or videoconferencing since they could receive ongoing feedback through these solutions, which leads to increased adherence to self-management.

Itoh et al. [70] delivered strengthening exercises and patient education therapy using a mobile messaging application named *Secaide* to people with CLBP. Their results showed a significant improvement in the symptoms of CLBP for the intervention group. However, this study did not indicate the effectiveness of therapeutic interventions on CLBP on work productivity. Lewkowicz et al. [71] conducted a systematic review to combine recent scientific literature on digital therapeutic care applications' impact on people with CLBP. They showed that the intervention group's functionality had increased, and they had lower pain levels. They also indicated that decision support solutions, such as personalized feedback messages, push notifications, and data-driven activity recommendations, benefit overall engagement with the application and increase participants' ability to self-manage their recovery process. Hogan et al. [72] evaluated early user experiences with *The Veterans Health Administration Pain Coach* mHealth application developed to support veterans with chronic pain by providing features such as tracking, daily diaries, and set reminders. They showed that the application might be helpful for the self-management of pain, e.g. increasing self-efficacy and reducing pain interference. However, the application's adoption among veterans in this evaluation was limited. Rintala et al. [73] overviewed the literature on self-management mHealth applications and their impacts on disability and pain levels in people with CLBP. The applications provided content such as personalization, monitoring daily life activities and physical activity, targeted home exercises (strengthening and stretching), mindfulness training, and education regarding CLBP. They found promising results in decreasing the levels of pain and disability in people with CLBP. Kerckhove et al. [74] investigated the feasibility and acceptability of a mHealth application called *eDOL* for patients with chronic pain. eDOL collects the patients' data (pain, anxiety, sleep quality). It provides a web interface for doctors to graphically visualize the summary of data provided by their patients for clinical and therapeutic monitoring. This study revealed that eDOL is highly feasible and acceptable for patients and their physicians. It leads to continuing the use of the application and increasing adherence to managing chronic pain, personalized therapeutic management, and better patient-physician partnership.

Lo et al. [15] assessed an artificial intelligence-embedded mobile application named *Well Health Mobile App* to self-manage back and neck pain. They found that by using the artificial intelligence (AI) system, users spent more time on therapeutic exercises, and their pain was generally reduced. Sandal et al. [75] investigated the effectiveness of the AI-based *SELFBACK* application. They showed that the patients who used SELFBACK to supplement routine care had less LBP-related disability at three months than those who received usual care alone. SELFBACK delivered weekly recommendations for physical activity, strength and flexibility exercises, and daily educational messages. Self-management recommendations were tailored to participant characteristics and symptoms. Usual care included advice or treatment offered to participants by their clinician. Anan et al. [76] indicated that the short exercises provided by the AI-assisted health program system operates through a mobile messaging application named *the AI-assisted health program* for 12 weeks led to more adherence to the exercises and significantly reduced both neck/shoulder and CLBP pain. Amorim et al. [77] conducted a systematic review investigating machine learning (ML) usage to rehabilitate CLBP. They showed that the supervised ML methods used in rehabilitation could help health professionals and LBP patients to manage their condition. This could cause people to feel more positive about CLBP and decrease work absenteeism, which could ultimately represent economic and social gains. ML is a promising tool for personalized rehabilitation as it contributes to a new paradigm of healthcare in which interventions are based on individual patient characteristics. They showed that studies used mobile applications to help patients self-manage their LBP remotely by providing them with educational material and exercise guides and monitoring their performance and adherence. This is a promising method to use in the telerehabilitation field.

Zahari et al. [78] investigated the effects of patient education on older people with CLBP. They showed that patient education effectively reduces pain, positively impacts disability and rehabilitation, and causes feeling positive among older people with CLBP. Mbada et al. [79] developed an animated cartoon-based self-care application named *ACBSC*, including information and guides on extension exercise protocols in the McKenzie method for people with CLBP. Participants reported that the application mostly affected mindfulness/meditation/relaxation, increased happiness/well-being, and led to feeling more positive while targeting physical health. Overall, it served as a practical mobile application for the self-management of long-term CLBP. Browne et al. [80] evaluated the utility of a *PainNavigator* application providing self-assessment, education, and goal-setting for CLBP management. They found clinical significance for the application and showed that it could be easily used to improve rehabilitation and self-management. Table 3 summarises researchers' perspectives on using various mHealth applications for self-managing CLBP.

**Table 3:** Summary of the features and benefits of various mHealth applications for self-managing CLBP from researchers' viewpoint.

Reference	mHealth solution	Features	Benefits
Chhabra et al. [65]	“Snapcare” application	Personalization and customization, Goal setting, Gamification	Adherence, Feeling good/positive/in control
Zheng et al. [66]	“Ding Talk” application	Physical activity, Education/advice	Adherence, Feeling good/positive/in control
Huber et al. [67]	“Kai” application	Yoga/meditation, Education/advice, Scheduling and reminders for exercise or behavioural change	Pain reduction, Adherence
Dobija et al. [68]	Different mHealth applications (review paper)		Self-management, Rehabilitation
Agnew et al. [69]	Different mHealth applications (review paper)	Patient-physician partnership	Adherence, Self-management
Itoh et al. [70]	“Secaide” application	Physical activity, Education/advice	Pain reduction
Lewkowicz et al. [71]	Different mHealth applications (review paper)	Personalization and customization, Scheduling and reminders for exercise or behavioural change	Pain reduction, Adherence, Self-management
Hogan et al. [72]	“The Veterans Health Administration Pain Coach” application	Activity tracking, Scheduling and reminders for exercise or behavioural change, Diary/self-assessment	Pain reduction, Self-assessment
Rintala et al. [73]	Different mHealth applications (review paper)	Activity tracking, Physical activity, Yoga/meditation, Education/advice, Personalization and customization	Pain reduction, Self-management
Kerckhove et al. [74]	“eDOL” application	Personalization and customization, Diary/self-assessment, Patient-physician partnership	Aherence
Lo et al. [15]	“Well Health Mobile App” application	Gamification, Artificial intelligence	Pain reduction, Adherence
Sandal et al. [75]	“SELFBAC” application	Personalization and customization, Education/advice, Scheduling and reminders for exercise or behavioural change	Pain reduction, Rehabilitation
Anan et al. [76]	“the AI-assisted health program” application	Physical activity, Artificial intelligence	Pain reduction, Adherence
Amorim et al. [77]	Different ML-based mHealth applications (review paper)	Physical activity, Personalization and customization, Education/advice, Scheduling and reminders for exercise or behavioural change, Artificial intelligence	Self-management, Rehabilitation, Feeling good/positive/in control
Mbada et al. [79]	“ACBSC” application	Physical activity, Education/advice	Self-management, Feeling good/positive/in control
Browne et al. [80]	“PainNavigator” application	Education/advice, Goal setting, Diary/self-assessment	Self-management, Rehabilitation

#### 3.1.3 Health professionals' viewpoint

This section will provide an overview of the existing literature on health professionals' perceptions of using mHealth solutions to support people with CLBP in self-management. In the following paragraphs, we will summarize the key findings from these studies and discuss their implications for future research. Integrating digital self-management solutions into healthcare demand the involvement of healthcare professionals in adopting and using the solutions as part of the care pathway. Since people with CLBP usually self-manage, health professionals should help by providing them with skills and knowledge. Self-management does not mean that people are alone in managing their health conditions. On the contrary, it empowers people to know when they need advice for diagnostic assessment and symptom relief [81]. Therefore, health professionals are critical in supporting people with CLBP to self-manage their condition.

Heuvel et al. [82] investigated the experiences of 14 physiotherapists in the Netherlands who provided self-management support to people with non-specific LBP. They demonstrated that physiotherapists believe that self-management is beneficial for people with non-specific LBP. Most participants expressed a need to better integrate self-management support in their treatment. They explained that they needed more knowledge (courses, knowledge clips), opportunities to exchange experiences (social support), and tools for patients (mobile application). A qualitative survey by Hutting et al. [83] suggested that physiotherapists and exercise therapists consider self-management support a robust method to manage the non-specific LBP. However, they showed that therapists mainly provide patient education instead of self-management support. Future research should investigate the effects of individualised self-management support as part of the regular treatment programs provided by physiotherapists, postural exercise therapists, and other healthcare providers. It should also include developing tools to assist healthcare providers in assessing patients' self-management skills and facilitating the patient-physician partnership.

Fu et al. [84] undertook a systematic review and realized that a partnership between patients and health professionals might support patients to self-manage their CLBP. They identified seven factors that affect patient-professional partnerships: communication, mutual understanding, roles of health professionals, information delivery, patients' involvement, individualized care, and healthcare service. Chala et al. [85] investigated the perception and conceptualizing of health professionals (medical doctors and physiotherapists) toward self-management for people with CLBP in Ethiopia. They found that self-management is a new concept for many of them, and they lack the competencies to provide self-management support for people with CLBP. Many of the support methods they proposed mainly focused on improving impairments rather than helping patients make self-efficacy skills. Therefore, there is a need to improve health professionals' self-management support competencies through training.

Guardado et al. [14] conducted qualitative research by interviewing 23 healthcare professionals from three countries with good healthcare quality indicators such as Finland, Spain, and Switzerland. They showed that more than half of healthcare professionals considered self-management mHealth solutions, and the data derived from them could benefit patients and healthcare professionals. They believed that the advantages of using patient-generated health data through activity tracking include knowing more about patient behaviours and activities, which could help healthcare professionals better understand factors affecting patients' health and how treatment could be adapted and thereby facilitate the patient-physician partnership. Kong et al. [86] investigated the perception and current use of 186 physicians in the USA toward mHealth solutions. More than half of them were willing to discuss mHealth solutions with patients. They showed that proof of accuracy and precision of collected biometrics through tracking and sensors is the most crucial metric that increases the likelihood of implementing information gathered by the mHealth solutions in their practice, e.g., to employ patients to better follow through in behavioural change and medication adherence.

Overall, studies showed that healthcare professionals have positive expectations about using self-management mHealth and patient-generated health data through automatic tracking or self-assessments and its possibilities for care. Table 4 summarizes health professional viewpoints toward features and benefits of using mHealth solutions to self-manage CLBP.

**Table 4:** Summary of the features and benefits of various mHealth solutions for self-managing CLBP from health professionals' viewpoint.

Reference	Method	Features	Benefits
Heuvel et al. [82]	Interviewing 14 physiotherapists in Netherlands	Education/advice, Scheduling and reminders for exercise or behavioural change, Goal setting	Self-management
Hutting et al. [83]	Qualitative survey on 38 physiotherapists and exercise therapists in Netherlands	Diary/self-assessment, Patient-physician partnership	Self-management
Fu et al. [84]	Systematic review on 10 papers	Education/advice, Patients-physician partnership	Self-management
Chala et al. [85]	Interviewing 24 medical doctors and physiotherapists in Ethiopia	Patients-physician partnership	Self-management
Guardado et al. [14]	Interviewing 23 healthcare professionals from Finland, Spain, and Switzerland	Activity tracking, Patients-physician partnership	Self-management, Reducing the burden on clinicians
Kong et al. [86]	Survey of 186 physicians in the USA	Activity tracking	Adherence, Feeling good/positive/in control

Further research is required to clarify how chronic patientś health data could be better interpreted, modelled, and presented, using scientific-based approaches, to be efficiently integrated into healthcare practices in ways that can make sense to healthcare professionals and can bring value to the patient-physician relationship in aspects such as monitoring, treatment, and shared decision making [14].

#### 3.1.4 Patients' viewpoint

This section focuses on patients' perceptions of using mHealth solutions to self-manage their CLBP. In the following paragraphs, we will provide an overview of the key findings from these studies, including patients' attitudes towards using mHealth solutions, the perceived benefits and barriers of mHealth use, and the factors influencing patients' intentions to adopt mHealth for CLBP self-management.

Patients are increasingly counselled to take responsibility for their own care. In a study by Kawi [20], the main themes derived from 110 participants' responses about how they self-manage their CLBP include taking medications, doing physical activity, making changes in lifestyle, utilizing heat and cold applications, resting, utilizing other physical and alternative methods. They showed that to achieve positive health outcomes and foster self-management behaviours, individuals require assistance from healthcare professionals. Alison et al. [87] investigated 27 studies with 487 participants. They identified the influencing factors for self-management of chronic musculoskeletal pain from the patient's point of view. They divided them into two categories: external factors like health care practitioner role, supportive environment, and accessibility and internal factors like physical factors, knowledge and understanding, and psychological factors. They showed that learning to self-manage requires continuous support from either healthcare professionals or social circles. Moreover, they illustrated that health professionals could improve self-efficacy by increasing patients' knowledge about their pain, employing goal setting, and finding ways to help them access continuous support from health professionals or through group programs. Al-Abbadey et al. [88] investigated the users' perspectives by reviewing a physical activity application named *MapMyRun* on Google Play. They identified eleven behaviour change techniques among the features of MapMyRun. They categorised them into two main themes: ‘Effort’ and ‘Self-monitoring.’ They indicated that the developer of future mHealth applications should focus on user-friendliness and social support or social features (communication with or competition among users, their family, and friends), as both may raise the chances of users' continued usage of the application.

Cooper et al. [89] showed that the CLBP patients who received primary care physiotherapy could be better assisted to self-manage their pain by being provided self-management education, traditional education, and patient-physician partnership support such as telephone calls, direct access, and review appointments. Understanding patients' motivations and needs are essential to ensuring higher acceptance of mHealth solutions [90]. Pornillos et al. [91] investigated people's perception of mHealth applications in the Philippines using the Technology Acceptance Model. They found that most respondents have a positive attitude towards using mHealth applications. Though, there was a considerable difference in the perception according to the type of lifestyle. Besides, mHealth users tend to perceive mHealth applications more positively than non-users. In line with it, Kheirinejad et al. [32] contrasted the viewpoints of people with chronic pains who adopted mHealth solutions and the expectations of those who have not. They showed that people who used mHealth solutions found them easy to use, reliable, and valuable. They also revealed that users had fewer privacy concerns compared to non-users. Instead, nonusers expected mHealth to facilitate interactions with health professionals. In contrast, the users felt that such connections do not exist.

Jezrawi et al. [12] conducted a study in Ontario on 94 patients interested in using mHealth applications. They showed that patients trusted recommendations and reviews from healthcare professionals and medical organizations when selecting applications. They also were motivated to continue using applications when they supported goal setting, tracking, data sharing, decision-making, and empowerment. Svendsen et al. [92] investigated the experiences of patients who participated in a randomized controlled trial of an application called *selfBACK*. They showed that most participants were satisfied and perceived benefits from implementing the selfBACK. They showed that fundamental factors for facilitating self-management are a friendly, motivational, and reassuring supporter, tailoring and personalization, convenience and ease of use, trustworthiness, perceiving benefits, and tracking achievements. They also showed that essential impeding factors for favouring self-management are functionality issues, suboptimal tailoring and personalization, insufficient time or conflicting life circumstances, not perceiving benefits, and inadequate involvement of healthcare professionals. Heuvel et al. [82] showed that physiotherapists believe self-management has an added value in treating people with CLBP. They found that most patients expressed a demand for more education (courses, knowledge clips), opportunities to exchange experiences (social support), and tools (applications) to better self-manage their pain.

Hunter et al. [93] investigated how mobile applications could help people self-manage CLBP by searching six databases and several non-academic sources, in addition to the 19 realist interviews conducted with patients. They showed that many people with CLBP rely on healthcare professionals' support because they do not know how to manage their symptoms. They indicated that a mobile application allows users to gain confidence and agency to manage their condition independently by providing them with knowledge, advice, and strategies to self-manage CLBP. They also indicated that monitoring their progress with a mobile application and sharing this data with a healthcare professional can help a person with CLBP convey more of a ‘complete picture’ of how they manage their condition and thereby improve the communication and quality of a healthcare consultation.

In general, users/patients had a positive perspective on using the mHealth applications to self-manage their CLBP. Table 5 summarized patients' viewpoints toward features and benefits of using mHealth solutions to self-manage CLBP.

**Table 5:** Summary of the features and benefits of various mHealth solutions for self-managing CLBP from patients' viewpoint.

Reference	Method	Features	Benefits
Kawi [20]	Examining the perception of 110 participants	Physical activity, Yoga-meditation, Scheduling and reminders for the exercise of behavioural changes, Patient-physician partnership	Self-management
Alison et al. [87]	Literature review on 27 studies and examining the perception of 487 participants	Education/advice, Goal setting, Patient-physician partnership, Social support	Self-management
Al-Abbadey et al. [88]	Examining 3253 users' reviews on “MapMyRun” application	Social support	Adherence
Cooper et al. [89]	A qualitative study on 25 participants	Physical activity, Education/advice, Patient-physician partnership	Self-management
Jezrawi et al. [12]	Examining the perception 94 patients on mHealth applications	Activity tracking, Goal setting	Adherence
Svendsen et al. [92]	A qualitative interview study of 26 participants on using “selfBACK” application	Activity tracking, Personalization and customization, Goal setting, Diary/self-assessment, Patient-physician partnership	Self-assessment
Heuvel et al. [82]	Patients' perception from physiotherapists' perspective	Education/advice, Social support	Self-management
Hunter et al. [93]	A literature review on 19 realist interviews on using mobile applications	Activity tracking, Educational/advice, Patient-physician partnership	Self-management

#### 3.1.5 Benefit score

The present study employs a weighted ranking system, as depicted in Table 6, to evaluate the features of the application. This ranking is based on the number of benefits that were reported in previous research conducted from the perspective of researchers, health professionals, and patients (as outlined in sections 3.1.2, 3.1.3, and 3.1.4). The result of this ranking system is referred to as the “benefit score,” which is intended to provide an indicator of the relative benefits of each feature. A high benefit score implies that a feature is more advantageous than others, while a low score indicates the opposite. For example, let us consider the “Activity tracking” feature. Its benefit score of 13 is computed from a total of two from “Pain reduction”, three from “Adherence”, five from “Self-management”, one from “Feeling good/positive/in control”, and two from “Reducing the burden on clinicians” (including the repeated citations). Conversely, the “Yoga/meditation” feature has a benefit score of only four when assessed using the same method. To summarize, the benefit score is a valuable metric for assessing the relative benefits of various application features based on previous research studies. It enables a clear and concise comparison of the features, facilitating the identification of the most useful features for users' requirements.

**Table 6:** Features of different mHealth solutions and their benefits for the self-management of CLBP.

Features	Benefits					
	**Pain reduction**	**Adherence**	**Self-management**	**Rehabilitation**	**Feeling good/positive/in control**	**Reducing the burden on clinicians**
**Activity tracking**	[72][73]	[12], [53], [86]	[14], [72], [73], [92], [93]		[86]	[14], [41]
**Physical activity**	[70], [73], [76]	[66], [76]	[20], [52], [73], [77], [79], [89]	[43], [77]	[66], [79]	[52]
**Personalization and customization**	[73], [75]	[65], [74]	[71], [73], [77], [83], [84], [92]	[75], [77]	[65]	
**Yoga/meditation**	[67], [73]		[20], [73]			
**Education/advice**	[44], [67], [70], [73], [75], [78]	[44]	[52], [73], [77], [79], [80], [81], [82], [83], [84], [87], [89], [93]	[75], [77], [78], [80]	[54], [66], [78], [79]	[41], [52], [54]
**Scheduling and reminders for exercise or behavioural changes**	[52], [72], [75]	[53], [67]	[20], [52], [72], [77], [82]	[75], [77]		
**Goal setting**		[12], [53], [65]	[80], [82], [87]	[80]	[65]	
**Diary/self-assessment**	[72], [81]	[74]	[14], [72], [80], [83], [92]	[80]		
**Gamification**	[15]	[15], [65]			[65]	
**Patient-physician partnership**	[40]	[74]	[14], [55], [83], [84], [85], [87], [89], [93]			[14]
**Social support**		[88]	[82], [87]			
**Artificial intelligence**	[15], [76]	[15], [76]	[77]	[77]	[77]	

Table 6 presents a comprehensive summary of the findings from various stakeholders' perspectives, including researchers, health professionals, and patients. The table provides an overview of the features that mHealth solutions could offer, the potential benefits of these features for CLBP, and the corresponding references that demonstrate these benefits.

In the next section, we will look at the landscape of mHealth solutions on application marketplaces and how they reflect the expectations of the three stakeholders identified in our methodology.

### 3.2 mHealth on current application marketplaces

For our second dataset, we searched the current Android and iOS application marketplaces for applications that can help manage CLBP. The search results often do not explicitly state whether applications are made for managing CLBP or chronic pain, but many could be seen as beneficial without explicitly saying so. Thus, the process includes subjective considerations. We were inspired by various studies that showed the relation of CLBP with exercise, physiotherapy and education. Therefore we used these and similar keywords to narrow down the search terms in marketplaces.

#### 3.2.1 Application search strategy

According to the reasons that could lead to CLBP, the solutions that could help to manage it, and the initial brainstorming and literature survey, we defined the following keywords to find applications related to CLBP: “back”, “back pain”, “backache”, “back care”, “back exercise”, “back therapy”, “low back pain”, “lower back” suggested by [94], “chronic pain”, “lumbago”, “lumbar”, “spinal pain”, “spinal”, “yoga”, “pain management”, “pain”, “posture”, “posture correction”, “posture tracking”, “physiotherapy”, “physical activity”, “exercise”, “activity”, and “tracking”.

We searched Google Play and App Store in September 2022. We used the Node.js google-play-scraper^1^ module to search Google Play and Python itunes-app-scraper^2^ for App Store. In total, we got 163 applications from both marketplaces. Both projects were initially designed for earlier versions of the application marketplaces, thus limiting the search results. More recent similar projects were not found that would match our needs or function more efficiently than the used versions. Both marketplaces currently limit the search results of individual searches, thus limiting the total number of applications.

#### 3.2.2 Google play results

First, we searched Google Play and obtained 30 results per keyword (other than two keywords that got 15 and 19). We combined all the data from the intended keywords and got a dataset with 724 applications with an average rating of 3. Removing the applications with a user rating of 0 or NaN leaves us with 490 applications. 29 Applications had a user rating of less than three out of five and were also removed. We then looked through the results of each keyword and removed 230 duplicates, resulting in 231 applications.

Finally, we manually scrutinized the description of each application in the dataset and removed applications that did not follow our requirements. As such, we removed applications that were not related to the intended keywords, such as “activity”, which was about controlling and managing the activities (application usage) in the smartphone, or “tracking” keywords that included other kinds of tracking like object tracking, parcel tracking, location tracking, social media usage tracking or personal or work tracking. For the keyword “lumbar,” the search mostly found lumber-related applications. In the end, after removing the unrelated applications, we got 132 applications.

#### 3.2.3 App store results

We searched App Store using the Python itunes-app-scraper-dmi package and found between 11-15 applications per keyword, which seemed to be the limitation of the current package version. The combination of keywords resulted in 355 applications in total. Then we manually filtered out 25 applications in categories such as “Health & Fitness”, “Medical”, “Lifestyle”, “Sports”, and “Education”. Like Google Play, we removed the applications with a user rating score of 0 (229 results left) and a rating score of less than three (202 left). 62 duplicates were removed next (140 left).

**Figure 1:**
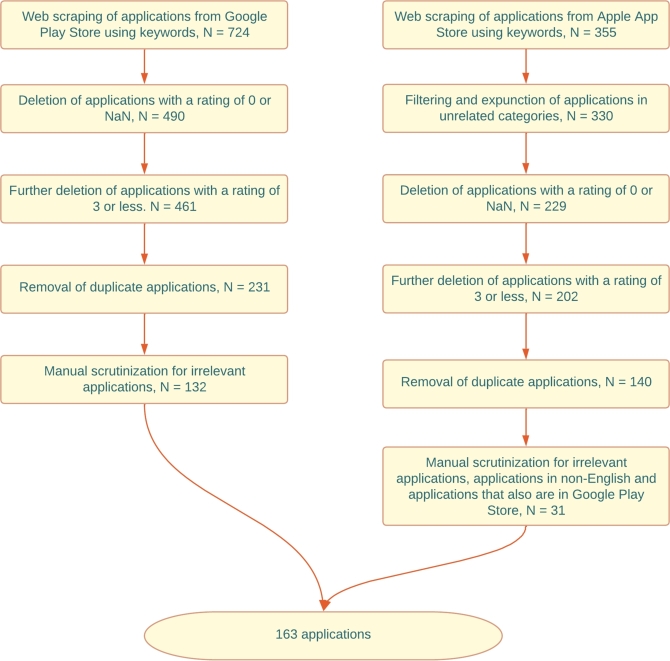
Steps of filtering the applications from Google Play Store and Apple App Store.

We then manually scrutinised the description of the remaining applications and removed those unrelated to our purpose. Some applications were removed since their language was not English (most were in German). Six applications were removed because those were also in the dataset scraped from Google Play. Some applications were unrelated, like abs workout for having “six packs”, and did not have other features like activity tracking or education that a person with CLBP would be eager to use. Some applications for managing pain using hypnosis were removed because none of our three stakeholders recommended hypnosis. In the end, we ended up with 31 applications from App Store. Fig. 1 shows all the steps of filtering the applications in both marketplaces.

## 4 Results and analysis

We review previous research and investigate the viewpoints of different stakeholders toward mHealth applications for managing CLBP. The following results analyse the application marketplace landscape and how it suits the needs of actual CLBP stakeholders at the time of this analysis in November 2022. The applications in our search process reflect those applications that end-users would find organically searching for methods for managing chronic pain.

We obtained a dataset of 163 applications from Google Play and the App Store. Our final dataset (obtained from merging two datasets from Google Play and App Store) includes “Price”, “User score”, “Name”, and “Description”. We manually reviewed the applications' descriptions to find details about the 12 features mentioned in Table 6, as well as two other aspects: whether the application was *developed by a medical expert* and whether it included *in-app purchases*. Based on the dataset of 163 applications collected from Google Play and the App Store, the average score rating of the applications is 4.36, and almost all are free. However, after reviewing the applications' descriptions, we noticed that Of the 163 applications, 62 (38%) explicitly mentioned they have in-app purchases in their descriptions, 12 (7%) mentioned that they are free, and 89 (55%) did not mention their pricing in the descriptions. Regarding development, 65 (39%) applications were developed by medical experts, while the remaining 98 (61%) did not mention their development team in the descriptions.

To better understand the viewpoints of different stakeholders toward the mHealth applications for managing CLBP, we analyzed the features and benefits offered by the applications in our dataset. The 12 features we focused on are listed in Table 6. These features were chosen based on their potential relevance to CLBP stakeholders and their availability in the applications included in our dataset.

Overall, our analysis of the mHealth application marketplace landscape indicates that various options are available for managing CLBP. However, the accuracy of the information provided in the descriptions of the applications, particularly regarding pricing, should be carefully considered by potential users. Additionally, the development team behind the applications may be an essential factor for stakeholders when choosing a mHealth application for managing their chronic pain.

**Figure 2:**
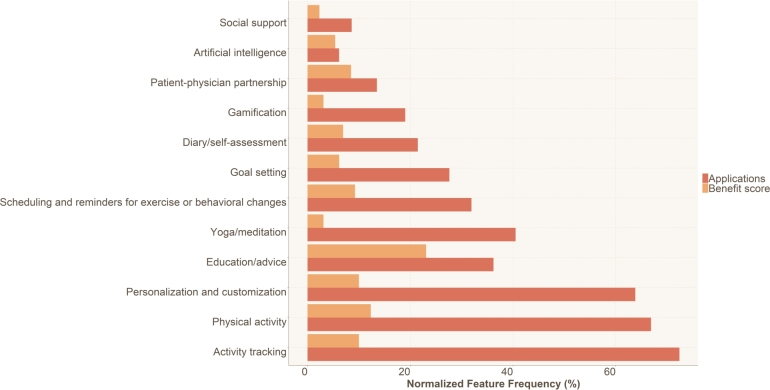
Frequency of features in the analysed applications and frequency of the references (articles) that show benefits for the features (benefit score) for managing CLBP.

In line with [95], [96], [97], [98], we reviewed the descriptions of each application by manually reading each application's description, extracted the features from the descriptions of the applications and investigated whether they contained features that were seen as beneficial by our three stakeholders, as listed in Table 6. Fig. 2 shows the percentage of applications that offer each feature and the benefit score of each feature (the percentage of studies that found those features beneficial in managing CLBP).

As shown, 72% of the applications provide “Activity tracking” features, while 64% provide “Personalization and customization” features. In contrast, only 10% of the studies we reviewed found these features beneficial for managing CLBP. Similarly, 69% of the applications offer “Physical activity” features, but only 12% of the studies found these features beneficial.

On the other hand, fewer than 10% of the applications provide features such as “Artificial intelligence” and “Social support”, and fewer than 6% of the studies found these features beneficial. In contrast, 23% of the studies found the “Education/advice” feature beneficial for managing CLBP, but only 36% of the applications offer this feature.

The most significant disparity between the percentage of applications that provide a feature and the percentage of studies that found that feature beneficial for managing CLBP was for the “Yoga/meditation” feature. 40% of the applications offered this feature, but only 3% of the studies found it beneficial.

Our analysis found that the least commonly provided feature was “Artificial intelligence”, and the least beneficial feature was “Social support”. We also detected two other features that some applications provided, such as a “Nutrition guide” (offered by 11.66% of the applications) and a “Set privacy level” (provided by 0.6% of the applications). However, the studies we reviewed did not discuss the benefits of nutrition in managing CLBP.

### 4.1 Over- and underrepresentation in marketplace applications

**Figure 3:**
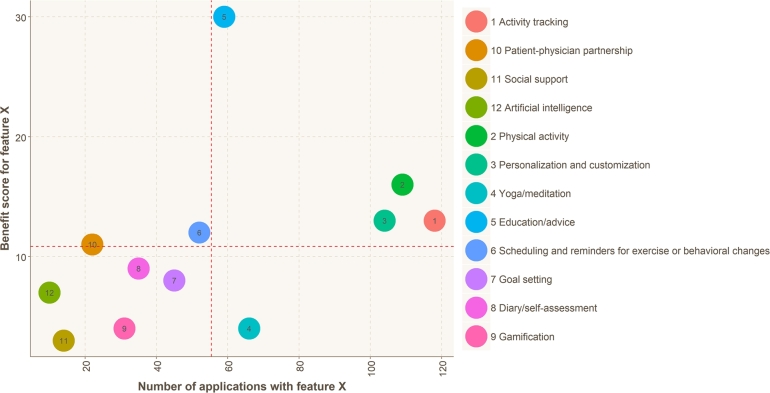
The ratio between the number of applications that provide different features and the number of studies that found those features helpful for managing CLBP (the weighted benefit score). The dotted lines represent the mean values for both ranges. Features higher on the y-axis are considered more beneficial.

Fig. 3 shows the number of applications that provide different features and that feature's benefit score. The four segments correspond to the over-/under representation of features in the application marketplaces (left-to-right on the x-axis) and whether those features are beneficial for managing CLBP or not (up-and-down on the y-axis). For example, the features in the lower right segment (#4 “Yoga/medication”) can be seen as unbeneficial and over-represented. Alternatively, the features in the top-left segment can be seen as under-represented but beneficial. This segmentation or categorisation can spark thought into understanding where application developers should focus on ensuring their applications are more beneficial for end-users.

### 4.2 Application categorisation using clustering

As a single application often provides multiple features instead of just one feature, the next step in the analysis was better understanding the similarities between different applications. For example, using the previous approach, we could consider yoga-related applications unbeneficial based solely on this one feature, even though, in reality, the same application could include other features, like “Activity tracking” or “Social support.” Thus, understanding similar application types offers more details about the overall landscape of current CLBP applications.

As the features are included in our dataset as a boolean value (each application either does or does not have a given feature), we use Hierarchical clustering and the HClust function from the fastcluster^3^ R package. We set the minimum number of clusters equal to our feature count (12), iterate in the range of [12,30] clusters and use a combination of the Elbow method, Silhouette method and Gap statistics with each cluster optimisation variable having equal balance in the decision to evaluate the cluster configurations. The optimal cluster configuration was ultimately found with 12 clusters, with all cluster configuration evaluation methods slowly declining for configurations above 12.

Table 7 shows the clusters' sizes and the four most common features that the applications in each cluster provide. It is worth noting that the size of clusters C2, C9, and C10 was one and were not included in further analysis. The majority of the applications are found in clusters C1 (N = 45), C3 (N = 66), C4 (N = 12), and C6 (N = 13), totalling 136 applications (83% of all 163). Each cluster was given a descriptive name that makes each cluster identifiable, according to the features most prominent in that cluster. The clusters (effectively application categories) are described in detail in the following:

**Table 7:** Feature distribution in clusters (C1, C3, etc.). Clusters C2, C9, and C10 had a size of one application and were not included.

	Size	1st ranked feature	2nd	3rd	4th	Name
**C1**	45	Physical activity (82%)	Education/advice (60%)	Yoga/meditation (33%)	Activity tracking (33%)	**Physical education**
**C3**	66	Personalization and customization (95%)	Activity tracking (86%)	Physical activity (63%)	Yoga/meditation (54%)	**Personalized tracking**
**C4**	12	Scheduling and reminders for exercise or behavioural changes (100%)	Activity tracking (100%)	Goal setting (83%)	Education/advice (83%)	**Tracking reminders**
**C5**	6	Patient-physician partnership (100%)	Education/advice (100%)	Personalization and customization (83%)	Physical activity (83%)	**Physician's advice**
**C6**	13	Diary/self-assessment (100%)	Activity tracking (69%)	Goal setting (38%)	Personalization and customization (30%)	**Tracking diary**
**C7**	6	Diary/self-assessment (100%)	Personalization and customization (100%)	Yoga/meditation (100%)	Activity tracking (100%)	**Personalized diary**
**C8**	5	Gamification (100%)	Yoga/meditation (100%)	Activity tracking (100%)	Goal setting (100%)	**Gamified yoga**
**C11**	3	Gamification (100%)	Activity tracking (100%)	Social support (33%)	Education/advice (33%)	**Gamified tracking**
**C12**	4	Personalization and customization (100%)	Gamification (100%)	Activity tracking (100%)	Social support (100%)	**Personalized social support**

**Physical education (C1)**: Applications in the Physical education cluster (C1) are primarily focused on providing users with tools for tracking and improving their physical activity levels. Most applications in this cluster (82%) provide the “Physical activity” feature, 60% provide “Education/advice”, and 33% provide “Yoga/meditation” and “Activity tracking”.

**Personalized tracking (C3)**: Applications in the Personalized tracking cluster (C3) offer a range of features for tracking and monitoring personal health and wellness. Most applications in this cluster (95%) provide the “Personalization and customization” feature, 86% provide “Activity tracking”, 63% provide “Physical activity”, and 54% provide “Yoga/meditation” features.

**Tracking reminders (C4)**: Applications in the Tracking reminders cluster (C4) are focused on providing users with tools for setting and achieving health goals, as well as reminding users to engage in healthy behaviours. All of the applications in this cluster provide “Scheduling and reminders for exercise or behavioural changes” and “Activity tracking”, and 83% provide “Goal setting” and “Education/advice”.

**Tracking diary (C6)**: Applications in the Tracking diary cluster (C6) are focused on providing users with tools for tracking and monitoring their health and wellness. Many applications offer diary or self-assessment tools for recording health-related information, such as symptoms, medication, and daily activities. All of the applications in this cluster provide “Diary/self-assessment”, 69% provide “Activity tracking”, 38% provide “Goal setting”, and 30% provide “Personalization and customization”.

The size of the rest of the clusters (C5, C7, C8, C11, C12) is smaller than 7. Clusters C7, C8, and C12 consist of applications that provide all the top three ranked features (100%).

#### 4.2.1 Significance of the clusters

**Figure 4:**
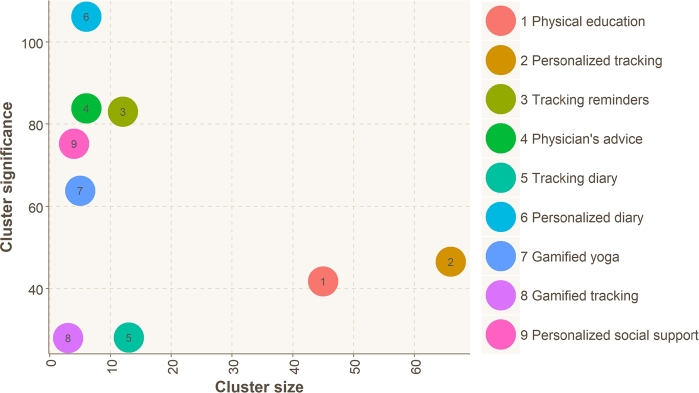
The ratio between the number of applications in each cluster (cluster size) and the significance of each cluster has been calculated by equation 1. Clusters higher on the y-axis are considered more significant.

In Fig. 4, we calculated the significance of each cluster using the equation (1).

(1) Significanceofthecluster=BenefitscoreofFeature1×PercentageofFeature1+BenefitscoreofFeature2×percentageofFeature2+etc.

As shown in Fig. 4, the “Personalised diary” cluster is the most significant. However, it only consists of six applications. The least significant clusters are the “Gamified tracking” and “Tracking diary”. The average significance for all the clusters is 61.82. The “Personalized tracking” and “Physical education” clusters are the most populated. However, the significance is less than average.

## 5 Discussion

People with disabilities or chronic pains perceive a tremendous advantage of staying at home and using mHealth applications [46]. However, many barriers still do not let different stakeholders take the maximum benefits from mHealth solutions. Barriers to creating the mHealth solutions are usually created by a lack of cross-disciplinary understanding, which can cause difficulties conceptualizing interventions, and unrealistic expectations [99]. The main limitation of using mHealth solutions by patients and health professionals is the lack of user-friendly applications, suitable user interfaces, high-quality designs, aesthetic metrics [69], lack of existing technology, concerns about regulation and efficacy of applications, security and privacy concern, compatibility with the workflow, lack of physician support, difficulty understanding the technology, health professionals' resistance to changing problems [100], insufficient knowledge and information about the mHealth solutions [57], the lack of trustworthy sources of informative applications [56], legal barrier, economic and financial factors, lack of health system policies, and lack of standards [100].

The following discusses the most to least significant features we found using their benefits score and frequency in applications. We also discuss the clustering of the applications. To ensure clarity and eliminate any potential ambiguity, we will provide precise definitions for the terms “benefits score,” “least significant,” and “most significant” before proceeding to the subsequent section.

• **Benefit score:** It is a valuable metric for assessing the relative benefits of various application features based on previous research studies (Table 6).
• **Least significant, Most significant:** We categorize a feature as “least significant” or “most significant” based on two criteria: its benefit score and frequency of occurrence across the applications examined in this paper.

### 5.1 The most significant features

Based on the benefit score and multiplicity of the features, “Physical activity” is a crucial feature for managing CLBP. Studies have shown that it is one of the most valuable treatments for improving disability and reducing pain in people with CLBP [101]. Therefore, CLBP application creators could include the “Physical activity” feature and make it more beneficial. Of course, if the feature is towards their aim and the application's purpose.

We also noticed a slogan in the description of one of the applications we reviewed: “*TRACK IT BETTER, TREAT IT BETTER.*” This slogan highlights the importance of tracking one's health and wellness to manage pain. “Activity tracking” is a feature that nearly three-fourths of the applications in our dataset provided. The benefit score is also greater than the average.

“Education/advice” is another essential feature, as gaining knowledge about one's pain and health can help individuals manage their condition. Many of the applications in our dataset offered educational resources, such as books, videos, and advice from health professionals, which can help people with CLBP learn more about their condition and how to manage it effectively.

Finally, “Personalization and customization” is a feature that has a good ratio between its frequency in the applications and its benefit score. People with CLBP often have different needs and preferences, and personalized and customizable treatment plans can help them manage their pain. This is why it is beneficial for mHealth applications for CLBP to offer features that allow users to tailor their treatment plans to their individual needs and preferences. Furthermore, mHealth application creators could provide people with CLBP information and advice (information about the nature of CLBP and encouragement to continue normal activities) tailored to their demands and abilities to help them self-manage their pain easier.

### 5.2 Neutral features

Slade et al. [102] conducted a review to identify and synthesize qualitative empirical studies investigating beliefs about exercise therapy for people with CLBP. They found that participants are likely to prefer and participate in exercise or training programs and activities designed to consider their preferences, circumstances, fitness levels, and exercise experiences. However, they would prefer supervised exercise over advice to exercise. People feel encouraged to exercise when they have regular contact with a healthcare professional and feel understood. In line with [32], [102], our analysis also shows that researchers, medical experts, and patients with CLBP expect patients to interact with their physicians using mHealth solutions. At the same time, users do not usually find such interaction available. Other studies have also found that users expect to interact with their physicians using mHealth applications, though few applications offer the “patient-physician partnership” feature [69], [72]. The reason could be inadequate knowledge and skills from application creators and a lack of collaboration between health professionals and application creators.

To make this happen, developers need to work with health professionals and ensure that mHealth applications are integrated into clinical practice. However, many challenges lead to a lack of collaboration between health professionals and application creators and also a lack of interaction between health professionals and patients. The reasons include healthcare professionals' lack of knowledge and confidence in using mHealth solutions [69], [103], a lack of existing technology [57], difficulty understanding the technology, health professionals' resistance to switching troubles [100], and lack of interest [46]. There are other reasons beyond the interest and knowledge of health professionals, like concerns about regulation and efficacy of applications, security and privacy concerns, compatibility with the workflow [100], lack of trustworthy sources of informative mHealth applications [56], legal barriers, economic and financial factors, lack of health system policies, and standards [100].

Educating health professionals makes them familiar with mHealth solutions. It assists them in the treatment process, like using patients' data for treatment, prescribing, and having partnerships through applications to help patients to be treated easier by self-managing their pain. It could benefit health professionals' businesses as well. A solution to familiarise health professionals with mHealth technologies, especially more advanced features of mHealth like artificial intelligence [104] is teaching mHealth technologies in medical education [105]. However, including mHealth courses in the medical curriculum might take time to become widespread worldwide.

Regular self-monitoring using mHealth applications can provide physicians with patients' data and leads to a better understanding of patients with chronic pain and improve treatment adherence [74]. Younger physicians at a lower professional rank, who tend to be more open to new technologies, are more likely to recommend mHealth solutions [72]. In countries where mHealth solutions are still in their infancy, it may be more effective to focus on the benefits of mHealth solutions for physicians themselves rather than the benefits for patients to increase adoption [106]. Future research and development of mHealth applications for CLBP should consider a scientific evidence base and engage in a multidisciplinary approach between application developers, healthcare professionals, and patients [16]. The future mHealth should also focus more on prevention and early disease detection rather than on remote assessment and treatment of patients [46].

Privacy concerns are also a significant barrier to the adoption of mHealth solutions, especially among individuals who have not used them before [32], [107]. To address this issue, independent authorities should certify applications to assure healthcare professionals of their ethical and data security practices. In addition, technical problems such as interoperability between data collection and medical records need to be addressed to limit the disruption to clinical work [108]. Clinical validation of mHealth solutions on a larger scale, rather than just pilot studies, is also needed to gain acceptance from patients and healthcare professionals.

Many applications in our dataset provided the Yoga/meditation feature. Still, few studies have investigated and found yoga applications beneficial in self-managing CLBP (i.e. the benefit score is low) - although it should be noted that yoga and medication alone are commonly seen as beneficial, similar to, e.g. stretching exercises. Further research is needed to understand the impact of Yoga/meditation provided by mHealth applications for self-managing CLBP. Despite being found beneficial in some articles, many applications did not offer the “Scheduling and reminders for exercise or behavioural changes” feature. This may be because developers want to focus on delivering fewer features to save time or lack the required knowledge.

In line with Zečević et al. [109], who analysed reviews of diet-tracking applications on Google Play. They found that, despite positive opinions from users, developers should pay more attention to technical issues and inform users about expected payments, along with refund and cancellation policies, to increase user loyalty. In this study, our data shows that most developers labelled their applications free and did not mention the in-purchase feature in the applications' descriptions. At the same time, the users need to pay for more advanced features. Developers should honestly express the features they provide for their applications and the level of facilities the users are provided based on the amount of money they pay. Users can only then choose the best applications based on their needs and budgets.

### 5.3 The least significant features

Few applications provide the “Artificial intelligence” feature, and its benefit score is also low. According to Esmaeilzadeh et al. [110], who studied how patients perceive the benefits, risks, and use of AI clinical applications for their healthcare, incompatibility with instrumental, technical, ethical, or regulatory values can be a reason for rejecting AI applications in healthcare. Thus, various risks are still associated with implementing AI applications in diagnostics and treatment recommendations for patients with both acute and chronic illnesses. Before the widespread roll-out of AI, more research is needed to identify the challenges that may raise concerns about implementing and using AI applications. Regulatory agencies should establish normative standards and evaluation guidelines for implementing AI in healthcare in cooperation with healthcare institutions.

Despite the numerous advantages of physical activities, the adherence level of patients' physical activity is not satisfactory [43]. To encourage users to continue using the application and increase adherence, applications can be designed based on gaming principles. For example, users can collect scores to upgrade levels and utilize a cognitive behavioural approach to promote regular exercise [15], [65]. Schmidt-Kraepelin et al. [111] found a positive relationship between the degree of gamification and user rating. However, few applications have adopted the “Gamification” feature for self-managing chronic pain, and studies have not found it beneficial in this context (it has low benefit score). Implementing gamification in mHealth applications could be challenging and require cooperation between health professionals, application developers, and game developers. Little is known about the extent of gamification in mHealth applications and whether it is worth the effort for developers to implement.

“Social support” is defined as support from a person with similar experiences. The advantages of social support include assistance in daily management, social and emotional support, linkages to clinical care and community resources, and ongoing support over time [112]. Multiple studies have demonstrated that social support interventions benefit self-managing chronic pain [113], [114]. However, application creators and researchers have not given much attention to this feature. The challenges of implementing the feature in applications could be the need for a secure platform, coordination with healthcare professionals, and the limited accessibility of this feature to patients in remote areas. Future research is needed to determine the significance of “Social support” in mHealth applications for chronic pain management.

Overall, various reasons are considered the barriers to mHealth adoption, which are related to lay people (lack of knowledge, necessary equipment, motivation, access to the devices, etc.), health professionals (lack of knowledge and training), technological (lack of user-friendly tools, ensuring the privacy, reliable internet, etc.) and organization (lack of integrated care policies, financial, etc.) [115]. While the studies indicate the users' concerns and doubts about their privacy [107], in this study, the feature that the developers paid the slightest attention to is “Setting the privacy level”. Most of them mentioned that we care about privacy. Yet, less than 1% provide a specific feature for privacy levels that enable users to determine the type and amount of data they are satisfied to share.

### 5.4 Examining clusters of features

The results of our analysis indicate that the most significant feature category is the “Personalized diary” cluster, which suggests that recording daily activities such as pain, sleep, and diet is beneficial for the self-management of CLBP. This is supported by the fact that the cluster contains the “Personalization and customization” feature, which shows that developers are aware of the need to customize and personalize the applications to meet the unique needs of individual users.

Another interesting finding is that the “Gamified tracking” cluster is the least significant, indicating a lack of attention from developers and researchers in this area - or that these methods are simply not found to be very beneficial. This is surprising, given that the cluster contains the “Gamification” feature, which has been shown to be effective in encouraging users to continue using the application and increasing adherence to treatment plans [116]. It is possible that the challenges of implementing gamification in mHealth applications for CLBP are too significant or that there is simply not enough interest in this area.

Overall, our analysis suggests that personalized and customized approaches are beneficial for integrating into mHealth applications for self-managing CLBP. Future research and development in this area could focus on developing and implementing personalized and customized features to improve the effectiveness of mHealth applications for self-managing CLBP. Additionally, further research is needed to understand the challenges and barriers to implementing “Gamification” in mHealth applications for CLBP and to determine how to overcome these challenges to improve their functionality.

### 5.5 Future work

Our initial findings from this paper can lead to multiple avenues of future work. The first step could be conducting a more extensive and more comprehensive study with interviews and surveys to investigate the functionality of mHealth applications for self-managing CLBP and comparing the results with this study, especially to understand the reasons behind the low benefit of the “Gamified tracking” cluster and exploring ways to improve it, and ways to improve the multiplicity of the “Personalized diary” category. This work could also lead to developing a framework for evaluating the benefit of mHealth solutions for self-managing CLBP and applying it to new and existing solutions. Lastly, it could lead to investigating the role of “Social support” and “patient-physician partnership” in self-managing CLBP and exploring ways to integrate these features into mHealth solutions so that they could benefit multiple stakeholders.

### 5.6 Limitations

In this study, we faced various limitations during the data collection, searching the papers, scraping the applications and analysing the data. The results from different perspectives are based on the studies we found and the research done so far. Other features might benefit the users, but there is insufficient research about them. Recently, Google Play Store does not load more than 30 applications searched by keywords on the first page. Accordingly, we could not scrape the information of more than 30 applications on scraping tools such as “ParseHub”, “Apify”, “Scrapy”, and “Scraper API” or by libraries in “Python” or “Javascript”.

Since downloading and installing every application was time-consuming, we reviewed the applications' descriptions and categorized the features explained there. The descriptions may be outdated. The applications might have other features that application creators might not have written in the descriptions, or the term mentioned in the description may not present the envisioned feature. Some applications added other features to their current version or added cost to the applications but did not mention it in the description. Some applications mention the application is free even in the description. Still, they had in-app purchases, making the price analysis challenging and inaccurate.

It is worth noting the keywords that we used for searching the applications in the two marketplaces are more comprehensive than the ones we used for searching the papers. The reason is searching on Google Scholar is based on the exact keywords. In that case, some keywords were irrelevant to using mHealth for chronic pain or CLBP, like “exercise” or “tracking”. In contrast, application marketplaces show the related applications to the keywords. The keywords even might not be in the title or description.

We acknowledge that our study has limitations that should be considered when interpreting the results. Our study is based on the available evidence. We also acknowledge that the terms “benefit score” and “significance” may not fully capture the potential benefits of mHealth applications.

## 6 Conclusion

This study aimed to provide valuable insights to mHealth application creators regarding the benefits of different features from multiple stakeholders' perspectives. In this study, we examined relevant scientific literature to determine mHealth application features and their benefits for self-managing CLBP from the viewpoints of three stakeholders: 1) researchers, 2) health professionals, and 3) patients. We then scraped and analysed available mobile applications for CLBP in application marketplaces such as Google Play Store and Apple App Store to assess whether the applications provide the features identified by the stakeholders and how common they are. We introduced a novel metric named “benefit score”, indicating a given application feature's aggregated gained benefits from all stakeholders. Based on the benefit score and multiplicity of the features in the applications, we showed that “Activity Tracking”, “Physical activity”, “Personalization and customization”, and “Education/ advice” are the most significant features as they are beneficial from the viewpoint of stakeholders and are represented by most applications. To understand what features usually come along with each other in the applications, we clustered the applications and estimated the significance of each cluster. We showed that the “Personalized diary” cluster, a combination of the “Diary/self-assessment” and “Personalization and customization”, is the most significant cluster. The “Gamified tracking” cluster, a combination of “Gamification” and “Activity tracking”, is the least significant cluster, indicating a lack of attention from application creators and researchers in this area.

## Author contribution statement

All authors listed have significantly contributed to the development and the writing of this article.

## Additional information

No additional information is available for this paper.

## Declaration of Competing Interest

The authors declare that they have no known competing financial interests or personal relationships that could have appeared to influence the work reported in this paper.

## Data Availability

Data will be made available on request.

## Footnotes

1 google-play-scraper.

2 itunes-app-scraper-dmi.

3 https://www.rdocumentation.org/packages/fastcluster/versions/1.2.3.
